# Gravitational Experimental Platform for Animal Models, a New Platform at ESA’s Terrestrial Facilities to Study the Effects of Micro- and Hypergravity on Aquatic and Rodent Animal Models

**DOI:** 10.3390/ijms22062961

**Published:** 2021-03-15

**Authors:** Julie Bonnefoy, Stéphanie Ghislin, Jérôme Beyrend, Florence Coste, Gaetano Calcagno, Isabelle Lartaud, Guillemette Gauquelin-Koch, Sylvain Poussier, Jean-Pol Frippiat

**Affiliations:** 1 Stress, Immunity, Pathogens Laboratory, SIMPA, Université de Lorraine, F-54000 Nancy, France; stephanie.ghislin@univ-lorraine.fr (S.G.); florence.coste@univ-lorraine.fr (F.C.); gaetano.calcagno@univ-lorraine.fr (G.C.); 2 Animalerie du Campus Biologie Santé, ACBS, Université de Lorraine, F-54000 Nancy, France; jerome.beyrend@univ-lorraine.fr (J.B.); isabelle.lartaud@univ-lorraine.fr (I.L.); sylvain.poussier@univ-lorraine.fr (S.P.); 3 Life Sciences in Microgravity, French National Space Agency, CNES, F-75001 Paris, France; guillemette.gauquelinkoch@cnes.fr

**Keywords:** gravity, spaceflight, development, adaptation, amphibian, mice

## Abstract

Using rotors to expose animals to different levels of hypergravity is an efficient means of understanding how altered gravity affects physiological functions, interactions between physiological systems and animal development. Furthermore, rotors can be used to prepare space experiments, e.g., conducting hypergravity experiments to demonstrate the feasibility of a study before its implementation and to complement inflight experiments by comparing the effects of micro- and hypergravity. In this paper, we present a new platform called the Gravitational Experimental Platform for Animal Models (GEPAM), which has been part of European Space Agency (ESA)’s portfolio of ground-based facilities since 2020, to study the effects of altered gravity on aquatic animal models (amphibian embryos/tadpoles) and mice. This platform comprises rotors for hypergravity exposure (three aquatic rotors and one rodent rotor) and models to simulate microgravity (cages for mouse hindlimb unloading and a random positioning machine (RPM)). Four species of amphibians can be used at present. All murine strains can be used and are maintained in a specific pathogen-free area. This platform is surrounded by numerous facilities for sample preparation and analysis using state-of-the-art techniques. Finally, we illustrate how GEPAM can contribute to the understanding of molecular and cellular mechanisms and the identification of countermeasures.

## 1. Introduction

Space is an adverse environment in which humans and animals face a combination of stressors (e.g., hypergravity during take-off and landing, microgravity throughout the flight, solar and cosmic radiation, confinement, isolation, sleep deprivation, disrupted circadian rhythm) that induce physiological dysregulations, such as muscle atrophy, cardiovascular dysfunction, bone demineralization, impaired cognitive processes, ocular problems, and reduced immunological competence [1].

Mechanical unloading elicits reductions in muscle mass, strength, and function, which upon return to Earth result in reduced function and performance [2]. An acute syndrome called cardiovascular deconditioning, associating orthostatic intolerance with syncope, and an increase in resting heart rate and a decrease in physical capability occur after spaceflight [3]. Microgravity has been shown to decrease heart rate and arterial pressure [4]. This was confirmed by another study reporting a misaligned diurnal rhythm of the heart rate during a flight [5].

Another major form of damage observed in humans and mice during spaceflight is bone loss due to an imbalance between bone formation and bone resorption [6,7,8,9,10]. Weight-bearing bones such as the femur, tibia, and vertebrae are more affected than non-weight-bearing bones. This might result in an increased risk of fractures [11]. Bone loss was also observed in zebrafish larvae subjected to simulated microgravity, whereas increased bone formation was noted when they were subjected to a 3-g hypergravity level [12,13].

The vestibular system, which functions to maintain body equilibrium, is affected by exposure to altered gravity [14]. Disruption of gravitational perception can lead to space motion sickness (pallor, cold, sweat, nausea, dizziness, vomiting), which is often felt transiently by astronauts. A decreased otolith-mediated vestibular response was noted after long-duration spaceflight in astronauts [15]. The vestibular system of fish is also affected, as it has been shown that otolith size and growth rate are increased under simulated microgravity [16]. Tadpoles that were flown shortly before the first appearance of the roll-induced vestibulo-ocular reflex (rVOR), a type of utriculo-ocular reflex that is specifically related to gravity, presented a depressed rVOR, whereas those launched after the first appearance of rVOR experienced an augmentation of that reflex [17], indicating that vestibular development is impaired by altered gravity [18].

Regarding the immune system, it has been demonstrated that dysregulation occurred and persisted during a 6-month orbital spaceflight [19,20]. A recent study revealed that approximately 50% of astronauts who spent six months onboard the International Space Station (ISS) faced immunological problems (e.g., infections, hypersensitivities) [21], thereby confirming in-flight dysregulation distinct from the influences of landing and readaptation following deconditioning [19,21,22]. Both the innate and adaptive compartments of the human and murine immune systems are negatively affected by space conditions (reviewed in [23,24,25,26]). The immune system of amphibians is also affected, as it has been shown that the production of their antibodies is altered [27,28,29]. Moreover, the use of tadpoles has led to the discovery of the perturbation of B-cell differentiation under spaceflight conditions [30], an observation that was confirmed in mice subjected to simulated microgravity [31] or real spaceflight conditions [32]. This observation was confirmed in cosmonauts [33].

These alterations in key functions show that spaceflight-induced physiological changes must be thoroughly investigated and understood to preserve astronaut health to safely and effectively accomplish the physically demanding goals of missions, especially during future deep-space exploration missions, such as the deployment of a lunar station, followed by multiple Mars flyby missions. These data also show that, in addition to mice, lower vertebrates, such as small fish species and tadpoles, can contribute to addressing human health questions. These animals are excellent models for developmental and biomedical research [34,35,36,37,38,39,40,41,42,43]. In addition, lower vertebrates meet many technical requirements associated with spaceflight experiments: their size and weight are reduced, many of them can be housed in a reduced volume, and they are easier to raise than mice.

Because space research is limited by access to the ISS, limited crew time, and various technical constraints, ground-based facilities are needed to investigate the effects of stressors encountered during space missions. Furthermore, ground-based facilities allow the isolation of single aspects of the space environment on Earth and make space research more accessible. They allow controlled experiments to be carried out with statistical power, in which the effect of one parameter (e.g., an alteration of gravity) can be analyzed without the interference of other parameters (e.g., radiation). Subjecting animals to ground-based models is invaluable in space research, as it can easily allow the evaluation of short-, medium-, and long-term effects.

In this paper, we present a new platform, which is part of the European Space Agency (ESA)’s terrestrial facilities and is accessible to the scientific community, for exposing amphibian embryos, tadpoles, or mice to modified gravity conditions, and we illustrate, using the immune and musculoskeletal systems as examples, how it can help to unveil molecular and cellular mechanisms developed in response to gravitational changes (see Figure 1 of Hariom et al. [1] for a recent summary of the effects of micro- and hypergravity on physiological systems). This platform can contribute to gathering useful information for the preparation of future manned space exploration missions through the analysis of mechanisms and signaling pathways preserved during the evolution of jawed vertebrates (gnathostomes). The availability of space hardware is of particular interest to prepare future space experiments (see Section 8 if you wish to use facilities of the Gravitational Experimental Platform for Animal Models (GEPAM) platform).

## 2. Equipment Available in the GEPAM Platform

### 2.1. Aquatic Rotors

**Figure 1:**
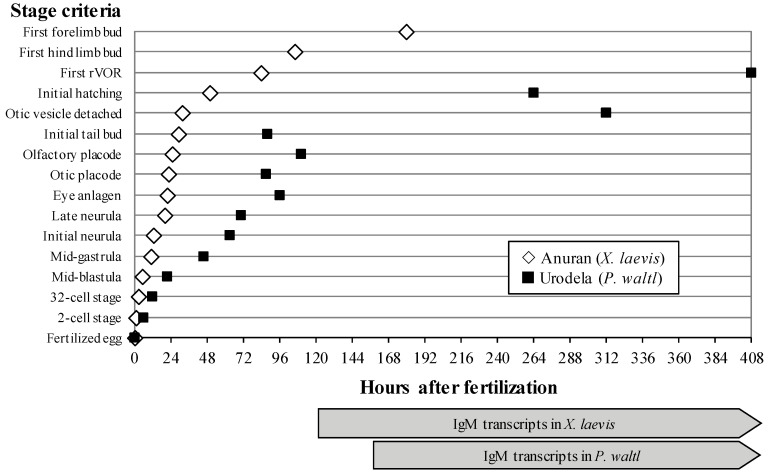
Comparison of anuran and urodela embryo developmental rates using *X. laevis* and *P. waltl* as examples. Gray arrows indicate the expression of IgM heavy-chain transcripts in both species. rVOR: vestibuloocular reflex. Adapted from [18,44] with the permission of Elsevier Ltd. and John Wiley and Sons.

Small aquatic animal models can perceive gravity changes, as evidenced by behavioral changes, alterations in bone formation, and in several pathways involved in bone, muscle, immune, or cardiovascular development [12,13,30]. Three aquatic rotors are available in the conventional sector of the Animalerie Campus Biologie Santé (ACBS, the animal facility of the biology and health campus) animal department to expose the embryos or tadpoles of four amphibian species (*Xenopus laevis*, *Xenopus tropicalis*, *Ambystoma mexicanum*, *Pleurodeles waltl*) to different levels of hypergravity. The species is chosen according to the biological process to be studied. Indeed, anuran embryos (*X. laevis* and *X. tropicalis*) develop much more quickly than urodela embryos (*P. waltl* and *A. mexicanum*) (Figure 1). Thus, urodela embryos can be used to study effects on processes occurring at early stages of development, whereas anuran embryos can be used to analyze effects on later stages of development.

**Figure 2:**
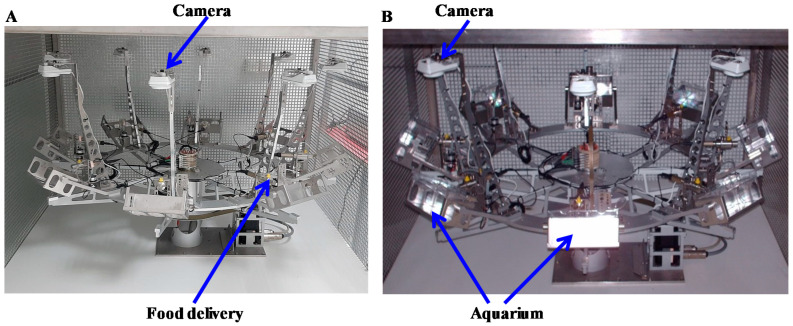
Rotor allowing the centrifugation of eight 0.5-L aquariums. (**A**) Centrifuge without aquariums in gondolas. (**B**) Centrifuge with the aquariums.

The first aquatic rotor (Figure 2) has eight gondolas, each accommodating an aquarium (155 × 85 × 74 mm) that can contain up to 0.5 L of water. Five levels of hypergravity are possible (1.5, 2, 3, 4, and 5 g). During hypergravity exposure, aeration of water by bubblers, food provision by a computer-controlled distribution system, temperature, lighting and ventilation are controlled. Furthermore, embryos/tadpoles are monitored by cameras operating both in visible and infrared light. The water level is checked regularly, and the water lost through evaporation is replaced.

**Figure 3:**
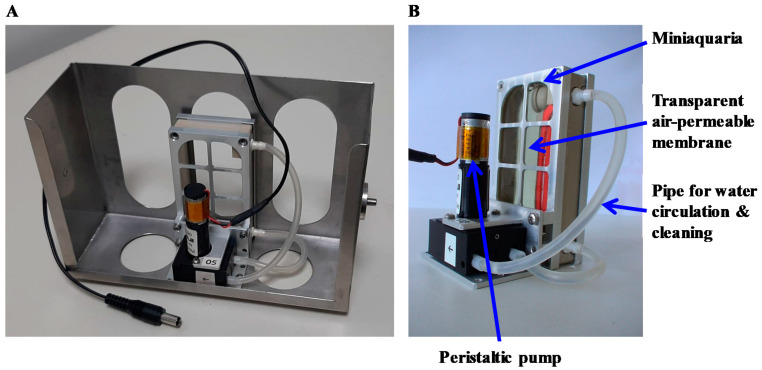
(**A**) Gondola of the rotor allowing the centrifugation of eight miniaquariums. (**B**) Miniaquariums have both large sides covered with transparent air-permeable membranes. These miniaquariums have a water circulation loop provided by a peristaltic pump with waste removal by an activated carbon filter and an osmotic pump that delivers the food. Each miniaquarium is 80 mm long, 40 mm wide, and 20 mm high.

The second aquatic rotor (Figure 3) can accommodate eight miniaquariums, allowing the development of amphibian embryos onboard the ISS up to developmental stages requiring feeding [45]. Five levels of hypergravity are possible (1.5, 2, 3, 4, and 5 g). Each miniaquarium contains 64 mL of water. Aeration is ensured by the fact that both large sides are covered by a transparent air-permeable membrane. These membranes, which are impermeable to H_2_O, prevent evaporation losses. In addition, each miniaquarium has a circulation of water provided by a pump that allows the disposal of waste by the passage of water through a filter. Food is gradually provided to the animals of each miniaquarium from a tank driven by an osmotic pump. Here, animal surveillance is visual and carried out daily. Temperature, lighting, and ventilation are controlled during hypergravity exposure.

**Figure 4:**
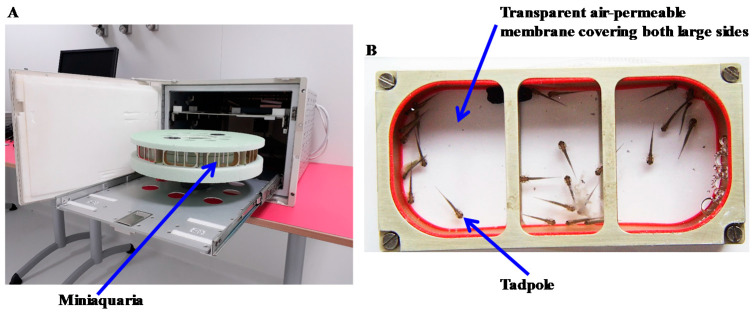
(**A**) Rotor allowing the centrifugation of 10 miniaquariums without a food supply and a water cleaning system. (**B**) Each miniaquarium is 80 mm long, 40 mm wide, and 20 mm high and has two transparent faces that are permeable to O_2_ and CO_2_ but impermeable to H_2_O. In (**B**), amphibian tadpoles can be seen.

The third aquatic rotor (Figure 4) allows us to subject ten miniaquariums to a hypergravity level of 3 g; these miniaquariums have been designed to allow the development of amphibian embryos onboard the ISS up to food intake. These miniaquariums are used to study the effects of altered gravity on early developmental stages in which food is not needed because embryos use their yolk as a food source. Each miniaquarium has a capacity of 64 mL of water. As with the other type of miniaquarium, aeration is provided by transparent membranes that are permeable to air but impermeable to water. Temperature and aeration are controlled in the room in which the rotor is located. There is no lighting system in the container housing this rotor. If lighting is required or if other levels of hypergravity are required, these miniaquariums without a food distribution system can be placed in the gondolas of the first aquatic rotor (Figure 2).

**Figure 5:**
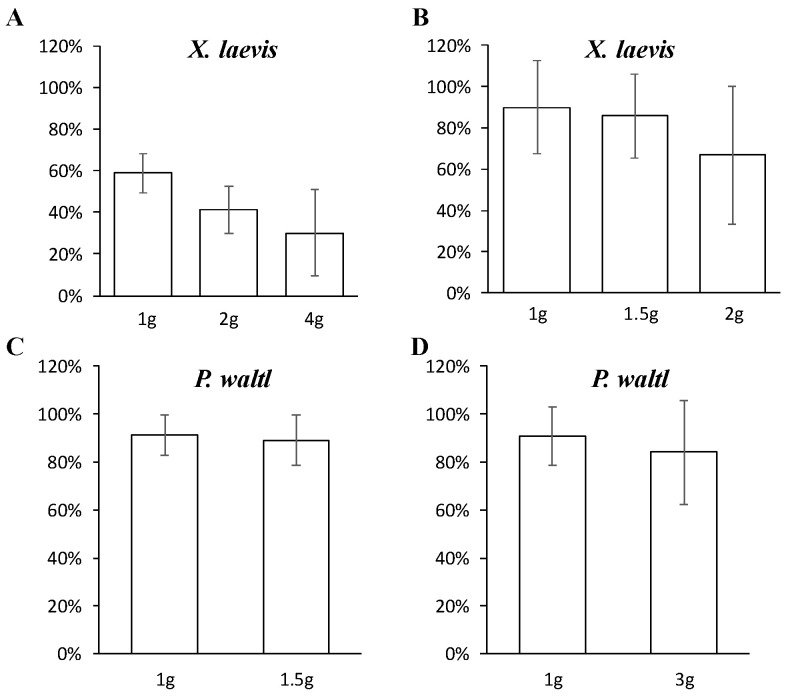
Examples of survival rates of anuran and urodela embryos, or tadpoles, raised under different levels of hypergravity for different durations. (**A**) Survival rates of *X. laevis* after 3 weeks of development at 2 or 4 g by comparison to 1 g controls. Embryos were at stage 8 of development at the beginning of the experiment (~5 h post-fertilization). (**B**) Survival rates of *X. laevis* after 2 weeks of development at 1.5 or 2 g by comparison to 1 g controls. Embryos were at stages 55–60 of development at the beginning of the experiment (~32–46 days post fertilization). (**C**) Survival rates of *P. waltl* after 10 days of development at 1.5 g by comparison to 1 g controls. Embryos were at stages 19–20 of development at the beginning of the experiment (~77 h post fertilization). (**D**) Survival rates of *P. waltl* after 10 days of development at 3 g by comparison to 1 g controls. Embryos were at stages 19–20 of development at the beginning of the experiment (~77 h post-fertilization). Aquariums containing 0.5 L of water and rotor 1 (Figure 2) were used to obtain the results presented in panels (**A**,**B**). Miniaquariums without a food supply and a water cleaning system and rotor 3 (Figure 4) or rotor 1 (Figure 2) were used to obtain the results presented in panels (**C**,**D**). No statistically significant differences could be detected. Lower survival rates are observed in panel (**A**) because embryos were not selected before initiating the experiment. Consequently, some unfertilized eggs were included.

Figure 5 shows good survival rates of anuran and urodela amphibian embryos or tadpoles raised under different levels of hypergravity using the devices present in the GEPAM platform. However, as shown in Figure 5A, if an experiment requires working with very early stages of development, it is necessary to carefully ensure correct fertilization before starting an experiment, as this may reduce the number of live tadpoles at the end of the protocol.

### 2.2. Random Positioning Machine (RPM)

**Figure 6:**
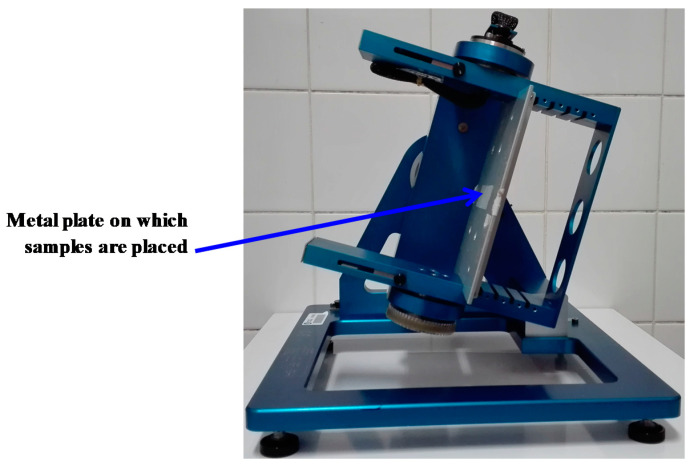
Picture of the random positioning machine (RPM), allowing the exposure of amphibian embryos or tadpoles to simulated microgravity.

A desktop random positioning machine from Dutch Space BV (Figure 6) is available to subject embryos, placed in miniaquariums without a food supply and a water cleaning system, to simulated microgravity. One to two miniaquariums can be mounted in this machine, which will randomly rotate them around the Earth’s gravity vector, resulting in an average net force close to zero, therefore simulating microgravity. Temperature, lighting, and ventilation are controlled in the room hosting this machine. This machine can also be placed in a cell culture incubator to subject cells to simulated microgravity.

### 2.3. Rodent Rotor

**Figure 7:**
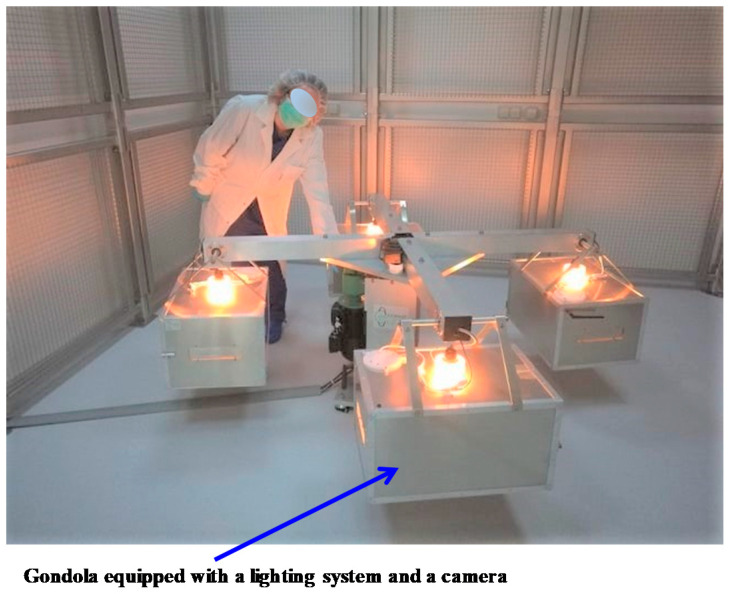
Large-radius rodent rotor allowing the exposure of mice to any desired g-level up to a maximum of 4 g.

A large-radius rodent rotor (Figure 7) is available in the specific pathogen-free sector of the ACBS animal facility. This centrifuge has four 55 × 38 × 30 cm (L × W × H) gondolas into which cages containing mice can be placed (typically four mice per cage). The speed can be adjusted to any desired g-level up to a maximum of 4 g. Mice can be supplied with enough food and water for three weeks so that the centrifuge can operate continuously during this period. Shorter exposures are possible, as well as longer exposures (>3 weeks after cleaning the cage, replenishing food and water). Each gondola contains an on-demand programmable lighting system and a camera (working in the visible and infrared light ranges) allowing remote control, day and night, of the mice in their cages. Environmental variables such as temperature, humidity, and ventilation are controlled in the room that houses this rotor. Four control gondolas are available. They are placed in a static position in the room containing the rotor to ensure that all environmental parameters, with the exception of the gravity level, are the same as in the centrifuge.

### 2.4. Cages for Mouse Hindlimb Unloading

Hindlimb unloading (HU) is a model frequently used to simulate the effects of microgravity [46]. Hindlimb-unloading cages (35 cm deep × 15 cm wide × 26 cm high), manufactured according to [47], are available in the specific pathogen-free sector of the ACBS animal facility. In these cages, mice are suspended using a dressing retention sheet wrapped around the tail and a wire hooked to a swivel-pulley system. The swivel pulley glides along two stainless steel rods that run the full length of the cage, providing a full 360° range of movement. The suspension angle is 25°–30°, so that only the forelimbs touch the grid at the bottom of the cage. Two types of controls can be performed: control mice housed in standard cages (35 cm deep × 15 cm wide × 14 cm high) and orthostatically restrained mice. In this last group, the mice are attached by the tail, but all four limbs are allowed to be in full contact with the floor of the suspension cage.

## 3. Housing Conditions

The ACBS animal facility (agreement number C54-547-30), with a surface area of 2700 m^2^, offers a high level of services and allows the breeding and maintenance of animals either under conventional conditions or in a rodent-specific pathogen-free area. ACBS meets the best technical standards in terms of housing and experimentation, in accordance with the European Community guidelines (2010/63/EU) for the use of experimental animals in compliance with the Replacement, Reduction, and Refinement (3Rs) requirements for animal welfare. ACBS is also involved in a quality assurance process attested by the obtention, in 2019, of the StAR-LUE label (Structure d’Appui à la Recherche—Lorraine Université d’Excellence (Research Support Structure—Lorraine University of Excellence)). This high-tech infrastructure can accommodate up to 3500 rodent cages and ~30 genetically modified mouse strains. Environmental variables such as temperature, humidity, ventilation, and pressure gradient versus corridors (in order to maintain animal health status) are controlled independently for each room of the murine and aquatic sectors.

**Figure 8:**
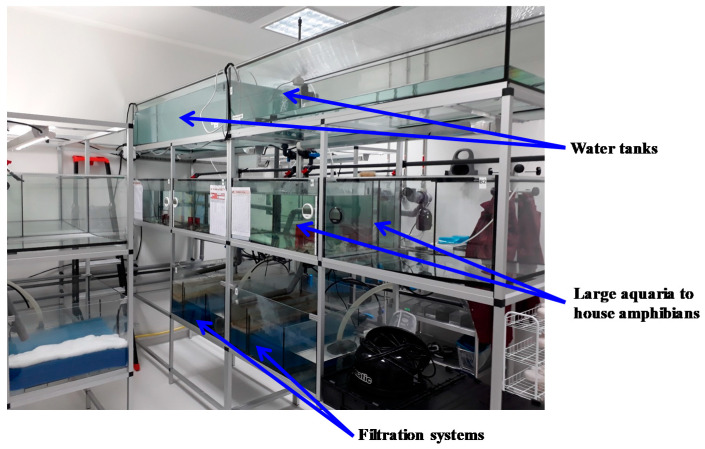
Amphibian housing area of the ACBS animal facility. Both adults and embryos/tadpoles can be reared in this sector under controlled conditions.

The amphibian housing area (Figure 8) is located a few meters from the aquatic rotors. Amphibian housing conditions meet the physiological and behavioral requirements of these species. Water quality is monitored twice a week. Temperature is monitored daily, as well as feeding behavior and the appearance of possible symptoms. In addition, food and water quality have been harmonized with those of a major producer of *X. laevis* and *X. tropicalis* in France to facilitate the acclimatization of the animals upon their arrival at ACBS.

## 4. Relevance to ESA’s Space Exploration Program

Exposing amphibians and/or mice to modified gravity can help answer several questions identified as of importance in ESA roadmaps (http://esamultimedia.esa.int/docs/HRE/SciSpacE_Roadmaps.pdf, accessed on 13 March 2021), such as:

How are the structure and function of cells and tissues influenced by gravity, and what are the gravity perception mechanisms? This will involve, for example, identifying changes in the mechanical properties of individual cells, tissues, and model organisms in response to altered gravity; assessing the effects of altered gravity on epigenetic, genetic and repair mechanisms; and studying stress responses induced by altered gravity conditions and their consequences.

How do gravity alterations affect animal systems at the cell or tissue level? This will involve studying the effects of microgravity and hypergravity at the molecular and cellular levels on the development, response, and regulation of the immune, musculoskeletal, cardiovascular, and central nervous systems and other physiological systems, such as gut microbiota, and tissue regeneration and healing in animal models.

How can an integrated picture of the molecular networks involved in adaptation to gravity changes in different biological systems be obtained? To answer this question, it will be necessary to compare different cell types in the same organism and/or a single cell type in different organisms, for example, by applying-omics approaches. Addressing this question is key to allowing the development of countermeasures.

## 5. How Can GEPAM Contribute to the Understanding of Molecular and Cellular Mechanisms?

There are numerous transgenic and mutant lines and strains of mice, *Xenopus* and zebrafish, as well as animal models of human diseases (see [48,49,50] for more details). GEPAM can allow the exposure of these various models to gravity changes from which cells and tissues can be extracted and analyzed on-site, using advanced molecular and cellular techniques (e.g., flow cytometry, cell sorting, imaging, epitranscriptomics, high-throughput sequencing, and proteomics (see [51])) to gain fundamental knowledge about the effects of gravity changes on physiological systems and their interactions. For example, immunocompetent cells are derived from hematopoietic stem cells (HSCs) that reside in the bone marrow within specialized niches made up of bone and vascular structures, and interactions between HSCs and their niches control the balance between quiescence, self-renewal, and differentiation of HSCs [52]; the sympathetic nervous system and the hypothalamic–pituitary–adrenal axis are strongly involved in the brain–gut axis that drives communication between the central nervous system and the gastrointestinal tract, including microbiota [53]. In addition, models of human disease can contribute to the understanding of the genetic and genomic basis of human biology, health, and disease.

**Figure 9:**
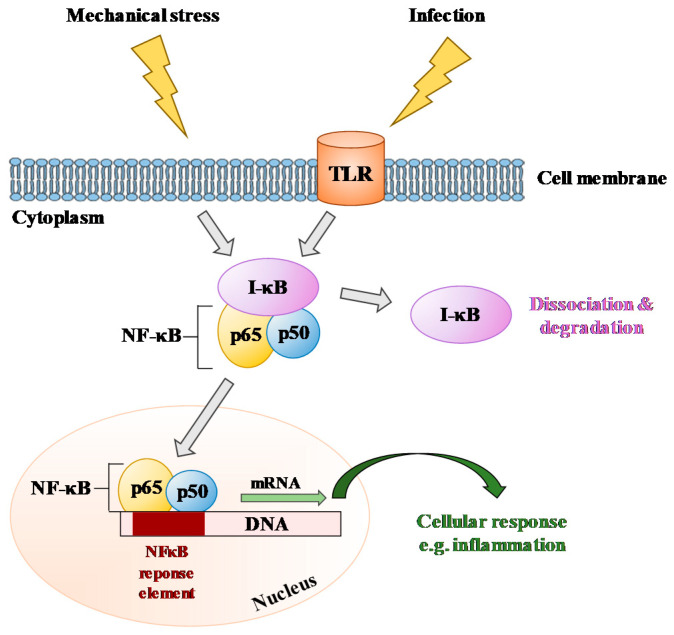
Schematic presentation of a major signaling pathway frequently affected in many different cell types of different species in response to altered gravity, mechanical stress, or infection. NF-κB: nuclear factor-kappa B; I-κB: inhibitor of κB; TLR: Toll-like receptor.

Here are some examples of results obtained using tools such as those of the GEPAM platform. Exposure of *Pleurodeles waltl* embryos to simulated microgravity (RPM) or hypergravity (3 g) until hatching revealed that the amount of IgM heavy-chain transcripts is higher at 3 g and lower at 10^−2^–10^−3^ g by comparison to 1 g controls [30]. A lower expression of Ikaros mRNAs, essential for establishing a lymphoid transcriptional program [54], was also noted in microgravity larvae, thereby suggesting that B-lymphopoiesis may be sensitive to gravity changes, an observation that was later confirmed in mice subjected to simulated microgravity [31] and real spaceflight conditions [32] and was also confirmed in cosmonauts [33]. An upregulation of NF-κB transcripts was also noted in hypergravity larvae, whereas a downregulation of these mRNAs was observed in larvae developed in the RPM, thereby showing that this signaling pathway is gravity-sensitive. The same conclusion was deduced from other studies, although the cell type determines whether the NF-κB pathway is activated or inhibited [55,56,57,58,59], as confirmed by a recent review [60]. These data show that NF-κB is commonly affected across many different cell types of different species under true or simulated spaceflight conditions (Figure 9). The adverse effect of spaceflight on B lymphopoiesis and the gravity sensitivity of the NF-κB pathway, involved in inflammation, T- and B-cell development, and responses to pathogens [61], may contribute to explaining the increased susceptibility to infection during space missions.

The development of *P. waltl* larvae under simulated microgravity or simulated microgravity and perturbed circadian rhythm also revealed modifications of the amounts of complement component 3 (C3) mRNAs and/or proteins, suggesting that complement expression can be modified under real spaceflight conditions, potentially increasing the risk of inflammation [62] and contributing to a better understanding of the inflammation observed during spaceflight [63,64,65,66]. More recently, Zhu and colleagues [67] showed that the retinoic acid-inducible gene (RIG)-I-like receptor (RLR) and Toll-like receptor (TLR) signaling pathways, essential for antiviral immunity, are significantly compromised in zebrafish embryos subjected to simulated microgravity. This discovery contributes to the understanding of the disruption of antiviral immune function in microgravity, which is manifested by an increased incidence of viral infections [68]. Indeed, the cardinal elements of the immune system are shared by all gnathostomes, including zebrafish and amphibians [69,70,71].

The proteomic analysis of *X. laevis* embryos exposed to simulated microgravity using an RPM during the first 6 days of development revealed that the expression of important factors involved in the organization and stabilization of the cytoskeleton, such as Arp (actin-related protein) 3 and stathmin, are heavily affected by microgravity [72]. In line with this observation, several other studies have reported that in various cell types, the cytoskeleton disorganizes in real microgravity or in response to altered gravity [73,74,75,76,77,78], thus showing that this structure, which gives shape and mechanical strength to cells, plays a role in sensing gravity changes, as suggested by Ingber [79]. Indeed, cells may sense mechanical stresses through changes in the balance of forces that are transmitted across transmembrane adhesion receptors that link the cytoskeleton to the extracellular matrix and to other cells [80,81].

The exposure of zebrafish larvae to 3 g from days 5 to 9 post-fertilization resulted in a significant increase in bone formation in a subset of cranial bones [12]. In contrast, 5 days of simulated microgravity caused a significant decrease in bone formation in zebrafish larvae [13]. In mice, quantitative PCR analyses have highlighted that the expression of two osteogenic differentiation genes (alkaline phosphatase (ALP) and type I collagen (Col-1)) is increased in the tibia when they are subjected to a hypergravity of 2 g for 2 weeks [82]. This deregulation was not observed after longer exposures at 2 or 3 g [83,84], suggesting a transient impact of hypergravity on bone metabolism factors and/or different impacts depending on the intensity of the applied gravitational force. Interestingly, the deregulation of these two factors was reverted by a vestibular lesion [78,80]. Vestibular lesion may moderate the effects of hypergravity, as it has been shown that hypergravity affects bone and muscle through vestibular signaling and subsequent autonomic nervous system in mice [83,85]. The hindlimb unloading model (HU), used to mimic microgravity, revealed activation of NF-κB, which plays a major role in bone mass reduction [10]. NF-κB1 has been shown to disrupt the proportion and/or potential of osteoprogenitors or immature osteoblasts [10]. NF-κB has also been shown to activate the expression of RANKL, which induces bone resorption [86]. Another important factor for bone homeostasis is Piezo-1. Studies have shown that Piezo-1 is a skeletal mechanosensor that regulates bone homeostasis and that its expression is reduced in osteoblasts of HU mice, which leads to bone loss and resorption [87,88]. HU also produced evidence of a decrease in ERK1/2 activity and an increase in p38 activation, leading to a decrease in the activation of RUNX2 and an increase in PPARγ2 activation, respectively. RUNX2 is an important factor for the differentiation of osteoblasts from mesenchymal precursors, and PPARγ2 is a factor involved in the differentiation of mesenchymal precursors into adipocytes. Thus, these changes lead to a decrease in osteoblast production in favor of adipocytes [89].

Recent studies performed using mice as animal models have shown that the adaptation of muscles depends on their function. Indeed, proteomic study of the soleus and extensor digitorum longus muscles—respectively slow and fast skeletal muscles—revealed different protein abundance profiles after 28 days spent at 3 g and suggested that the soleus is more sensitive to hypergravity than the extensor digitorum longus muscle [85]. In accordance with these observations, another report showed that the expression of MyoD, Myf6, and myogenin mRNAs (involved in myogenesis regulation) is increased in the soleus of mice that spent 4 weeks at 3 g and that, as observed in muscle, these deregulations are corrected by a vestibular lesion [83]. Later, the same team discovered that through the vestibular system, hypergravity enhances the expression of FKBP5 and OFLM1 genes in muscle [90,91]. The muscle response also depends on the applied gravitational force, as protein synthesis and phosphorylation of anabolic markers (AKT, p70s6k, 4E-BP1, GSK-3beta, and eEF2) were not modified in the soleus of mice exposed for 30 days at 2 g, whereas these parameters were altered in the tibialis anterior [92]. The duration of altered gravity exposure is another important parameter, as it was shown that MyoD mRNA expression was not affected after 2 weeks spent at 2 g, whereas it was decreased after 8 weeks spent at 2 g [84].

Finally, different studies have been conducted to elucidate the potential implication of humoral factors in muscle and bone communication when gravity is changed. In this context, it was noted that hypergravity elevates serum and mRNA levels of follistatin, an endogenous inhibitor of myostatin, in the soleus of mice. This increase was positively correlated with trabecular bone mineral content. Furthermore, the amount of follistatin mRNA was decreased in myoblastic C2C12 cells under simulated microgravity, suggesting the implication of follistatin in adaptation to gravity [82]. More recently, DNA microarray studies identified Dickkopf (Dkk) 2, a Wnt/β-catenin signaling inhibitor, as a potential humoral factor involved in muscle and bone communication. Indeed, HU significantly elevated serum Dkk2 levels and Dkk2 mRNA levels in the soleus of mice, whereas hypergravity significantly decreased those Dkk2 levels. In contrast to follistatin, the Dkk2 serum level was negatively correlated with trabecular bone mineral density [93].

Note that modifications of bone microstructure can impact the immune system. Indeed, although multipotent hematopoietic progenitors were not affected by HU [31], a decrease in red blood cells was observed in the bone marrow [94]. An HU-induced decrease in B lymphopoiesis was observed as of the common lymphoid progenitor stage, with a major block at the pro-B to pre-B cell transition. This observation was associated with a decrease in bone microstructure, a reduced expression of B-cell transcription factors (early B-cell factor (EBF) and Pax5), and an alteration in STAT5-mediated IL-7 signaling [31]. This blockage likely explains the lower percentage of B-cells observed in the periphery [94,95]. HU also decreased the ratio between helper and cytotoxic T-cells in the spleen [95] and increased the percentages of monocytes and macrophages in the bone marrow [94]. These last deregulations may be due to changes in the expression levels of hematopoietic-related genes such as leptin, GM-CSF, Flt-3, IL-3, and PPARγ2 [90].

## 6. How Can GEPAM Contribute to the Identification of Countermeasures?

Artificial gravity has been proposed as a countermeasure to mitigate physiological deconditioning caused by prolonged exposure to weightlessness [96,97,98] and therefore to protect human health during long-duration deep space missions. As mentioned in the introduction, immune dysregulation and bone loss are major adverse consequences of spaceflight. Indeed, spaceflight leads to a decrease in murine bone microstructure, hematopoiesis, and B-lymphopoiesis, as observed in old mice, suggesting that adaptation to space has immunosenescence characteristics [32,63]. Preservation of bone structure through chronic mild exposure to hypergravity could contribute to maintaining immune cell homeostasis. This hypothesis is supported by various studies showing that 2 g exposure has positive effects on several murine bone parameters [84,99] and that hypergravity increases bone formation in zebrafish [12]. Furthermore, the occurrence of allergy-related dysfunctional states associated with health problems in crews in space and analogous environments [25,100] could possibly be modulated by hypergravity [101]. Indeed, a recent study showed that hypergravity enhances the effect of dexamethasone in a murine model of allergic asthma and rhinitis, as indicated by the reduction of eosinophils in bronchoalveolar lavage fluid and eosinophilic infiltration into the lungs and nasal cavity [102]. Thus, combining drug administration with hypergravity exposure could potentially be a strategy to improve drug efficacy. It has also been shown that hypergravity has positive effects on trabecular bone and muscle typology in osteoarthritic mice [103]. These effects were similar to those induced by resistance exercises, but negative effects were noted for cortical bone. The latter observation shows that further studies are warranted to precisely determine how to use artificial gravity as a countermeasure. The intensity and duration of hypergravity exposure will need to be finely tuned to avoid side effects. Indeed, deleterious effects on the skeleton have been observed in mice after 3 weeks spent at 3 g [99]. A rupture of organism adaptation has also been observed at 3 g that leads to an increase in stress [104], which may have a negative impact on bones and the immune system [105,106]. Significant changes in intestinal microbiota, which is essential for survival, health, and well-being, as it ensures important functions and modulates the immune system, were also reported [107,108,109]. Furthermore, it has been shown that hypergravity exposure during murine development strongly modifies the repertoire of T-cell receptors (TCRs) of newborn mice [110] and that prolonged centrifugation (>1 day at 2 g) can affect the permeability of the blood–brain barrier [111].

Hindlimb unloading has been useful in identifying promising pharmacological countermeasures to mitigate the negative effects of spaceflight (reviewed in [112]). This ground-based model revealed that nucleotide supplementation has immunoprotective effects and lowers plasma corticosterone, which is a strong immune modulator. Hindlimb unloading also highlighted the immunoenhancer properties of the active hexose-correlated compound (AHCC). AHCC is an extract prepared from cocultured mycelia of several species of Basidiomycete fungi. AHCC increases the expression of the linker for activated T-cells (LAT) gene. LAT is the primary activator after TCR engagement. As an adaptor protein, the main function of LAT in TCR signaling is its tyrosine phosphorylation and subsequent recruitment of other signaling proteins. Upon TCR engagement, the phosphorylation of LAT allows it to interact with several SH2 domain-containing proteins, such as Grb2, Gads, and PLC-γ1. Thus, AHCC, through its action on LAT, can restore T-cell activation in immunosuppressive scenarios. AHCC also reduces inflammatory markers and stress hormones in hindlimb-unloaded rodents.

## 7. Conclusions and Perspectives

The Gravitational Experimental Platform for Animal Models, GEPAM, allows the exposure of aquatic animal models (currently amphibian embryos/tadpoles) and mice to altered gravity. This platform will contribute to examining the molecular and cellular mechanisms responsible for the manifold changes occurring in animals when exposed to altered gravity. Lower vertebrates, such as small fish species and tadpoles, are excellent models for developmental and biomedical research and meet many technical requirements associated with spaceflight experiments. Analyses of mechanisms and signaling pathways preserved during the evolution of jawed vertebrates are of particular interest, as they will provide very useful information for the preparation of future manned space exploration missions. This platform can also help validate certain hypotheses and experiments before their verification or implementation in real spaceflight conditions. The exposure of animals to altered gravity is also a means of seeking reliable and effective countermeasures. Testing nutrients and pharmacological products will provide new preventive and therapeutic tools to counterbalance dysfunctions encountered in space and on Earth, such as those induced by aging, as a multitude of similarities between the physiological deconditioning induced by spaceflight and that related to aging have been noted [113,114], or acute and chronic stress exposure [115]. In the future, developments allowing the exposure of small fish (e.g., zebrafish) or fish embryos to different levels of hypergravity or simulated microgravity are foreseen, as well as the installation of small cell culture incubators in gondolas of the rodent rotor.

## 8. Accessibility

GEPAM is accessible through ESA’s continuously open research announcements website [116]. Before applying, it is recommended to contact either J. Bonnefoy (julie.bonnefoy@univ-lorraine.fr), S. Ghislin (stephanie.ghislin@univ-lorraine.fr) or J.-P. Frippiat (jean-pol.frippiat@univ-lorraine.fr) to obtain more information, such as technical details.

## Data Availability

The data presented in this study are available in this published article.

## Abbreviations

3Rs	Replacement, Reduction and Refinement, a rule for performing more humane animal research
ACBS	Animalerie du Campus Biologie Santé (animal facility of the biology and health campus).
AHCC	active hexose-correlated compound
ESA	European Space Agency
g	gravity on Earth
GEPAM	gravitational experimental platform for animal models
HU	hindlimb unloading
ISS	international space station
RPM	random positioning machine
rVOR	roll-induced vestibulo-ocular reflex
StAR-LUE	Structure d’Appui à la Recherche—Lorraine Université d’Excellence (Research Support Structure—Lorraine University of Excellence)
TCR	T-cell receptor
TLR	Toll-like receptor
